# Horses Used for Educational Purposes in New Zealand: A Descriptive Analysis of Their Use for Teaching

**DOI:** 10.3390/ani10091547

**Published:** 2020-09-01

**Authors:** Lauréline Guinnefollau, Erica K. Gee, Elizabeth J. Norman, Chris W. Rogers, Charlotte F. Bolwell

**Affiliations:** 1 School of Veterinary Science, Massey University, Palmerston North 4442, New Zealand; E.K.Gee@massey.ac.nz (E.K.G.); C.W.Rogers@massey.ac.nz (C.W.R.); C.Bolwell@massey.ac.nz (C.F.B.); 2 College of Sciences, Massey University, Palmerston North 4442, New Zealand; E.J.Norman@massey.ac.nz; 3 School of Agriculture and Environment, Massey University, Palmerston North 4442, New Zealand

**Keywords:** horse, practical instruction, teaching, veterinary science, welfare, workload

## Abstract

**Simple Summary:**

Many equine and veterinary science degree programmes use horses during practical teaching classes. The use of horses during teaching was studied over a calendar year. The teaching horses were characterised as older non-reactive mares and geldings that had been used for teaching for a number of years after retirement from (harness) racing or sport. These horses were grouped into and managed as different herds based on suitability for specific practical teaching classes. The frequency of teaching activities per horse was relatively low (1–2 sessions per week). Two broad types of practical classes were identified which were characterised by the restraint method used (yards vs. stocks), duration of the class, and number of students per horse. The classes included rectal examinations (in stocks, shorter duration, few students) and general animal handling and husbandry, which included handling, lameness evaluation, clinical examination and foot trimming (in yards, longer duration, more students). Although the workload from teaching within this cohort of horses was low, more work to determine additional markers of teaching horses’ welfare may be required.

**Abstract:**

Horses are used in practical teaching classes in many equine and veterinary science degree programmes to develop and refine the handling and clinical skills of students. In this study, the activities of 24 teaching horses grouped in three herds were investigated over an entire calendar year. Although also used for research and general husbandry, teaching-related activities were the predominant use of the horses. Herd B was used for a greater number of teaching sessions (median = 28, IQR = 27–29.5 per year) than herds M (median = 21, IQR = 20–21 per year) and T (median = 19.5, IQR = 13.75–25.5 per year), which translates to a relatively low workload (one or two weekly sessions during the teaching semester). Sedation was used in dentistry classes (in alignment with national best practice standards) but was rarely required for other teaching activities. Mare reproductive rectal- and medical rectal examination practical classes (specific to 5th-year veterinary teaching and characterised by more restraint (in stocks)) were significantly shorter and had fewer students per horse than the other practical classes. Although the low workload reported suggests an opportunity to increase students’ exposure to horses without compromising the horses’ welfare, further investigation to determine specific stressors to the horses in the teaching environment may be required.

## 1. Introduction

Animal handling is a critical skill for veterinarians to master. The proper handling of horses is likely to minimise their stress and pain, lower their reactivity, and reduce the risk of injury to both the horse and handler [1,2,3,4,5,6]. Good animal handling skills depend not only on the knowledge of the animal’s behaviour and ethological needs, but on adequate and well-timed reactions to the animal’s behaviour changes that require experience and practice to develop [7]. In conventional horse riding, studies have started to evaluate the amount of experience required to substantially decrease the number of injuries (i.e., approximately 100 h of riding experience) [8], but these metrics are currently lacking to assess how much equine exposure is required to develop competency in horse handling. While veterinarians’ handling competency is often judged anecdotally by the clients through the horse’s behaviour and easiness to handle [2,3], there are limited data in the literature quantifying this skill.

Hands-on experiences with animals are therefore emphasised by most universities in their equine and veterinary science degree programmes [2] to complement theoretical teaching. The importance of practical classes in the curriculum has grown, as most students that now enter animal-based programmes come from an urban background with limited animal experience [9,10,11,12]. Equine practical teaching classes may well be the first occasion for some students to interact with a horse [13,14]. Successful and positive experiences (e.g., “getting it right”, “being good at it”) in the early stages of their riding careers have been reported to be major drivers in the development of elite equestrian riders [15]. This suggests that allowing students to become confident and competent veterinarians may be influenced by the type of early experiences they have with horses. To enhance students’ training, it would be useful to identify which type of activities should be provided to veterinary students and which are the most suitable horses for this purpose.

In the veterinary teaching environment, horses are used for a range of practical classes (e.g., animal handling, lameness investigation, rectal examination, dental examination and treatment) [1,11,13,16,17,18,19,20,21] in which students show various levels of equine experience and confidence with horses [12,22]. Horses are selected for each type of practical teaching class based on their behavioural characteristics and their apparent ability to cope with their use [11,16,18,21]. Therefore, the type and number of interactions with equine and veterinary science students are likely to vary between animals. These student-horse interactions themselves might also be inconsistent because of variation in the equine behaviour knowledge and equine experience of students [12,22]. A failure of students to correctly identify horses’ behavioural cues and to give an appropriate response at an appropriate time may lead to confusion for the horse [23,24,25]. As a result, horses may develop conflict behaviours that may negatively influence their welfare [23,26].

Despite the widespread use of horses for teaching purposes within universities, very little information is available describing the nature and frequency of these activities within the equine and veterinary science curriculum. The aim of the present study was therefore to describe and quantify the type and frequency of use of teaching horses over an entire educational year in a veterinary school in New Zealand.

## 2. Materials and Methods

The study population included twenty-four horses, used for undergraduate equine and veterinary science practical teaching classes, at Massey University, New Zealand. The horses included 3 Thoroughbreds, 19 Standardbreds, 1 Stationbred (crossbred) and 1 Kaimanawa (New Zealand feral horse). Ten horses were geldings and 14 were mares, with a mean age of 15 ± 4 years (one horse’s age was not officially recorded but thought to be over 15 years). The horses were pasture-based throughout the year, and for ease of management were kept in three herds of 7 (herd B–mares and geldings), 8 (herd T–mares and geldings) and 9 (herd M–mares only) individuals. The composition of the herd was based on the temperament and tolerance of the horses to the different teaching procedures. Quiet horses tolerant of naïve students comprised one herd (herd T) and were used for 1st-year practical handling classes. A second herd (herd B) was used for medical rectal examination classes, and a third herd (herd M) was used for mare reproductive rectal examinations. Pasture was the primary feed source, and in the winter months hay was also provided once daily at 2 (1–4) kg of dry matter/horse/day (median, interquartile range). Water was provided ad libitum from water troughs in the paddocks. The mean paddock size was of 1.9 ± 0.7 ha, and herds were rotated between paddocks based on availability.

The use of the teaching horses was studied over the 2018 calendar year (from 1st January to 31st December). At Massey University in the southern hemisphere, most teaching is concentrated into two semesters which follow the calendar year, but some 5th-year veterinary teaching continues outside the normal semester system. Teaching semesters were 12 weeks long separated by a 2-week mid-semester break. In 2018, semester 1 lectures were given from February 26th (week number 9) to June 1st (week number 22), with a mid-semester break from March 30th to April 13th (week numbers 14–15). Semester 2 lectures were given from July 16th (week number 29) to October 19th (week number 42), with a mid-semester break from August 27th to September 7th (week numbers 35–36).

This study retrospectively analysed data collected and stored electronically within an MS excel spreadsheet and the weight scale proprietary database (tru-test.com) whenever the teaching horses were brought into the teaching facility. The data recorded included the horses’ name and weight, herd of animal, date, length and type of event, name of main teaching staff involved, course and animal ethics committee protocol numbers, number of students in practical teaching class, use and type of medication (if administered), and additional comments during use.

Data were extracted and categorised. The date of the event was categorised by semester, while ‘calendar year’ referred to both semesters 1 and 2 and semester breaks. The type of use of the horse during an event was categorised as ‘teaching’ (i.e., horses used for practical teaching classes with students), ‘general husbandry’ (i.e., general husbandry care for the horses provided by staff members or veterinarians), ‘research’ (i.e., horses used for a research project–no change to management) or ‘other’ (horses used for neither of the above procedures, such as blood harvesting). Each teaching event was categorised based on the type of practical teaching class (i.e., ‘animal handling’, ‘clinical examination’, ‘foot trimming’, ‘lameness evaluation’, ‘dental training’, ‘medical rectal examination’ and ‘mare reproductive rectal examination’). Within an event, the use of each horse was identified as a ‘horse session’ (i.e., one session = one horse). The number of horse sessions varied between events. In teaching events, some classes required the use of the whole herd (e.g., medical rectal- and mare reproductive rectal examinations) and others only a selected portion of the herd (e.g., a maximum of four horses per animal handling practical class). The duration of each individual horse session was quantified as a ‘horse hour’ (e.g., 30 min = 0.5 horse hour).

If the number of students working with each horse was not recorded, then the number of students per horse was estimated by dividing the number of students in the practical teaching class by the number of horses used for this class. If medication was used, it was classified into three categories: ‘sedation’, ‘anti-inflammatory’, and ‘antibiotic’.

When used for teaching, the horses were individually kept either in stocks or in teaching yards. Information on the conduct of practical teaching classes, including the location of the horse during the class, as well as the type of restraint used, were obtained from class supervisors or technical staff members. Data were categorised according to the horse’s location during the practical teaching class in relation to conspecifics, the method of restraint, the year of students involved in the class, the number of students per horse, and the consistency of routine of the practical teaching class (Table 1).

### Statistical Analysis

Horse use data (name, herd, date, type of use, type and length of practical teaching class, use of medication, course number and number of students) were examined using pivot tables to generate frequency counts and percentages. The percentage of horse sessions involving the use of sedation for teaching purposes was calculated.

Differences in the uses between herds were tested because some practical teaching classes were herd-specific. Associations between the herd and the number of horse sessions in each category (i.e., teaching, general husbandry, research, other) were investigated using chi-squared tests. The distribution of the number of horse sessions was nonparametric, so differences between herds were tested using a Kruskal-Wallis analysis of variance. When required, multiple comparisons between herds were then tested for using a Dunn test (package *FSA*). Between types of practical teaching classes, a Kruskal-Wallis nonparametric analysis of variance (and Dunn test if necessary) was also performed to compare the length of horse sessions, and the number of students per horse. Statistical analyses were performed using R software version 3.5.3 (R Core Team, Vienna, Austria). The threshold used for statistical significance was *p* < 0.05.

The particularities of the practical teaching classes were then investigated using the variables available in Table 1.

## 3. Results

### 3.1. General Activity

From January to December 2018, the horses were used for a total number of 2091.5 horse hours. This included 1276.5 horse hours (534/1208 horse sessions–44.2%) of teaching, 250 horse hours (272/1208 horse sessions–22.5%) of general husbandry, 515 horse hours (337/1208 horse sessions–27.9%) of research (i.e., two behavioural and one physiological research projects) and 50 horse hours (65/1208 horse sessions–5.4%) of other uses. Teaching represented a total of 479.5 horse hours (herd B), 423.5 horse hours (herd M) and 373.5 horse hours (herd T).

Except for the involvement of herd T in research (7 horse sessions), the distribution of activities between herds in semester 1 did not differ. However, there was a difference in semester 2 between herds (Table 2). The proportion of teaching was different across the three herds, with a greater proportion of use for research with herd M (43.4% of horse sessions) than herd B (21.3% of horse sessions). The distributions of general husbandry activities and other uses were similar between herds.

### 3.2. Teaching Activity

**Figure 1:**
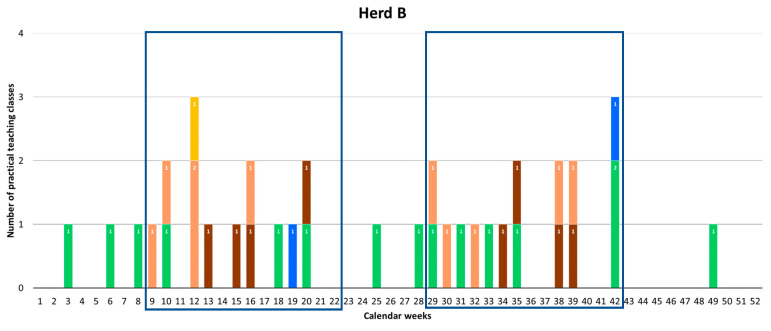

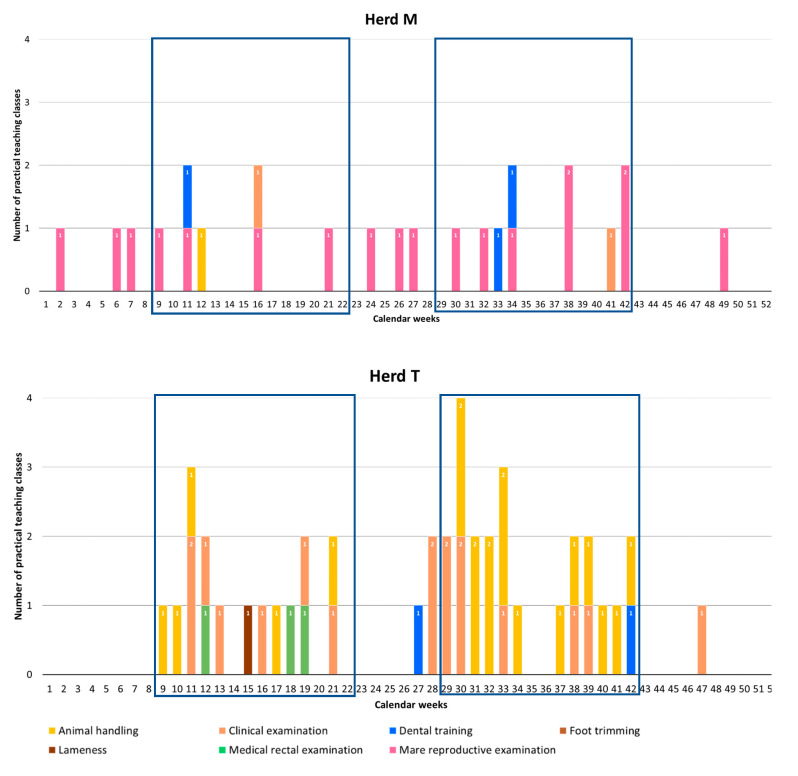
The numbers and types of practical teaching classes per week over the calendar year, i.e., any class for which a minimum of one horse was used for teaching per week, for each herd (B, M, and T). The blue boxes refer to the teaching semesters one and two, i.e., to weeks 9 to 22, and to weeks 29 to 42, respectively.

For each herd, Figure 1 shows the temporal use over the calendar year as the number of practical teaching classes per week. The majority of the use of horses for teaching occurred during either teaching semester one or two. Seventeen practical teaching classes were scheduled outside of the teaching semesters. Fourteen of these classes referred to 5th-year veterinary teaching and three classes were from courses for which start and finish dates differ from the normal University semester dates (Massey University website).

**Figure 2:**
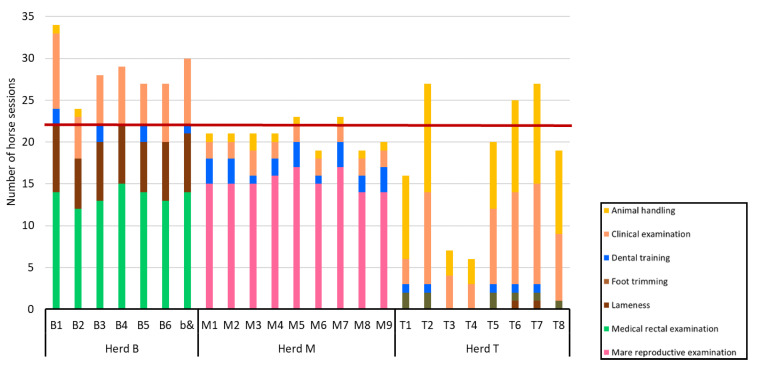
Individual numbers of horse sessions stratified by herd over the calendar year. The red line represents the median (=22) number of horse sessions of the three herds.

Over the calendar year, there were more horse sessions for herd B (median = 28, IQR = 27–29.5) than for both herds M and T (median = 21, IQR = 20–21, z = 3.17, *p* = 0.005; and median = 19.5, IQR = 13.75–25.5, z = 2.8, *p* = 0.01, respectively). Although there was some variation in the number of horse sessions, all horses of herd M were used for the same four types of practical teaching classes (Figure 2). In herd B, three horses did not take part in dental training classes, and only two horses were used for animal handling classes. The number of horse sessions was the most variable within herd T, with two horses rarely used. In addition to being used less frequently, these two horses were never involved in foot trimming, dental training, and lameness practical teaching classes (Figure 2).

Horses were rarely sedated for teaching purposes (7%, 35/534 horse sessions). Sedation was always used in dental practical classes (33/33 horse sessions), rarely for clinical (1/95 horse sessions) and medical rectal examinations (1/127 horse sessions), and never recorded for the following practical teaching classes: animal handling, foot trimming, lameness evaluation and mare reproductive rectal examination.

In mare reproductive rectal examination and medical rectal examination practical classes, the length of the horse sessions (Kruskal-Wallis test: χ^2^ (6) = 263.6, *p* < 0.001) and the number of students per horse (Kruskal-Wallis test: χ^2^ (6) = 217.0, *p* < 0.001) were significantly lower than in animal handling, clinical examination, lameness evaluation, foot trimming, and dental practical classes (Table 3).

### 3.3. Main Practical Teaching Classes

Over the calendar year, the four main practical teaching classes in terms of volume (i.e., number of horse sessions and number of horse hours) were mare reproductive rectal examination, clinical examination, medical rectal examination, and animal handling (Table 4). The rest of the teaching volume was divided between dental training, foot trimming, and lameness evaluation.

The use of the three herds was different between types of practical teaching classes. Herd B, herd M, and herd T were used, respectively, mainly for the following practical teaching classes: medical rectal examination (46.8%), mare reproductive rectal examination (73.4%), and animal handling (47.6%).

## 4. Discussion

Despite the widespread use of horses for educational purposes in most equine and veterinary science degree programmes around the world, there is a current lack of empirical information on the animals’ specific use. To the authors’ knowledge, this is the first study to quantify the use of horses for educational purposes in the teaching environment. Using a retrospective method over an entire calendar year, the present work documents in detail the management and teaching-related activities of a cohort of teaching horses.

Practical teaching classes are faced with many constraints (e.g., staff, budget, scheduling) in a tight curriculum [17]. The use of the horses is therefore dictated to a large extent by the programme and the challenge of providing sufficient time for students to practice their skills. Despite a more frequent use during the second teaching semester, the number of weekly teaching-related activities and hours use with these horses was low compared to the frequency and duration of activities (such as riding and training) in other horse populations and equestrian activities. Competition horses (i.e., dressage, show jumping, eventing) are trained for three to six 45–50 min sessions per week of varying intensity [27,28,29,30,31]. Similarly, studies reported approximatively one to three hours of work per day and at least one rest day per week for riding school horses [32,33,34]. In these populations, a description of the workload is often used for management purposes and to evaluate the amount of exercise-related physiological stress experienced by the animals. Authors usually report information on the intensity of the session (e.g., speed, duration, heart rate and other physiological measures) in addition to the frequency [28]. In this context, practical classes could be considered to provide a limited physical challenge to the horses. However, the parameters used to describe the intensity of the workload of ridden horses are not applicable to teaching horses whose movements are generally restrained during practical classes while students practice and improve their handling and clinical skills. Instead, in this population of horses, the frequency and regularity of teaching use may be suitable parameters to assess the workload of teaching horses [35].

The teaching horses and the herd composition had very few changes in the last 10 years and the age of the teaching horses was heavily skewed to the right, with many horses in their teens and a few in their early twenties. Given the long-term tenure of the horses, the stability of the herds and the low frequency of use, the authors hypothesise that this population of teaching horses may experience limited physiological or behavioural stress. For these cohorts of horses, given the nature of the teaching classes and the frequency of horse use, any behavioural stressors are likely to be limited to handling and interactions with inexperienced students. When they enter the veterinary programme, students have a poor understanding of equine behaviour and knowledge of the principles that constitute the way horses learn (i.e., learning theory) [12,22]. Associative learning (i.e., classical and operant conditioning) requires the use of at least one stimulus and aims to increase or decrease the frequency, duration, or intensity of future occurrences of desirable or undesirable behaviours [36]. If unable to correctly identify equine body signals, students are therefore unlikely to provide consistent cues and to apply appropriate signals, which may negatively reflect on their interactions with the horses. Mistimed and/or inconsistent signals can induce subsequent confusion and conflict behaviours and have been reported to increase arousal and reactivity levels in horses [3,25,26,36].

Students’ equine skills competency increased throughout the equine and veterinary science degree programmes. However, many students have very limited equine experience and low confidence in horse handling at enrolment, and some still show low self-assessed equine skills and confidence in 4th year [12]. Given the current low use reported for the teaching horses in this study, there could be an opportunity to increase student exposure to horses and horse handling. This could be implemented through either formal (syllabus) or informal opportunities. Some limitations with this proposal are the lack of data on the exposure hours required to achieve horse handling competence in naïve students, the suitable metrics needed to quantify horse handling competence and how often the horses could be involved in practical teaching classes. Without suitable evaluation criteria and metrics, it is difficult to model the ideal rate of use required to achieve optimal teaching outcomes.

Through the inherent content of practical classes, the advancement of the students in the programme and the class size capacities, different horses will encounter variations in the type and number of interactions with students. To accurately describe the potential impact on the horses’ welfare, further research is required on the interactions between horses and students to provide more robust descriptors of the relative workload these horses experience in the teaching environment. Investigation of other potential stressors during practical work is also warranted. Future studies could include direct measures of stress using behavioural and physiological indicators (e.g., recording of heart rate during practical sessions) of the horses while they are involved in teaching classes to determine additional markers of welfare.

## 5. Conclusions

This was the first study to describe teaching-related activities of horses kept for educational purposes. Although variable between and within herds, a relatively low frequency of teaching use was reported. Practical classes were different in their frequency and routine, in the number of students per horse and year of students, and in the location and restraint of the horses. Given these findings, there may be an opportunity to increase the use of the horses for teaching to optimise the value of the teaching experience for the students. However, for animal welfare considerations, more research is required to evaluate the optimal number and frequency of equine practical teaching classes.

## Figures and Tables

**Table 1:** Variables chosen to describe the practical teaching classes.

Variable	Categories
Location of the horse	Regular (i.e., same neighbour and location each time) or variable (i.e., different neighbour and/or location)
Year of students	1st, 2nd, 3rd, 4th or 5th year
Number of students per horse	
Length of horse session	
Method of restraint	High (i.e., horse in stocks with restriction of movements) or low (i.e., horse in a yard and held by a handler)
Routine of the practical teaching class	Regular or variable

**Table 2:** The numbers and percentages of horse sessions (i.e., teaching, general husbandry, research and other) for each of the three herds during the 2018 calendar year, and the 1st and 2nd teaching semesters. *p*-values were obtained from chi-squared tests when comparing the distribution of variables between herds B, M, and T.

	Herd B (*n* = 7 horses)	Herd M (*n* = 9 horses)	Herd T (*n* = 8 horses)	*p*-Value
Calendar year				
Teaching	199 (52.7%)	188 (41.1%)	147 (39.4%)	0.0003
General husbandry	77 (20.4%)	93 (20.4%)	102 (27.4%)	0.027
Research	83 (22%)	147 (32.2%)	107 (28.7%)	0.004
Other	19 (5%)	29 (6.4%)	17 (4.6%)	0.49
Semester 1				
Teaching	62 (63.9%)	61 (64.9%)	50 (53.2%)	0.19
General husbandry	35 (36.1%)	30 (31.9%)	36 (38.3%)	0.65
Research	0 (0%)	0 (0%)	7 (7.5%)	0.0007
Other	0 (0%)	3 (3.2%)	1 (1%)	0.16
Semester 2				
Teaching	99 (61.9%)	76 (41.8%)	85 (50.9%)	0.001
General husbandry	18 (11.3%)	17 (9.3%)	27 (16.2%)	0.14
Research	34 (21.3%)	79 (43.4%)	48 (28.7%)	<0.001
Other	9 (5.6%)	10 (5.5%)	7 (4.2%)	0.81

**Table 3:** Characteristics of each type of practical teaching class during the 2018 calendar year. The length of the horse session and the number of students per horse are presented with the median and interquartile range (IQR).

	Location of the Horse	Year of Students	Number of Students Per Horse	Method of Restraint	Routine of the Practical Teaching Class	Length of Horse Session (Hours)
Animal handling	Variable	1st, 2nd, 3rd, 4th	3 (2–4)	Low	Variable	2.5 (2.5–3)
Clinical examination	Variable	1st, 2nd, 3rd, 4th, 5th	5 (4–6)	Low	Regular	3 (3–3)
Dental training	Variable	3rd, 5th	2 (2–8)	High	Regular	3 (2.5–4)
Foot trimming	Variable	2nd, 4th, 5th	4 (2–3)	Low	Regular	3 (2–3)
Lameness evaluation	Variable	2nd, 3rd, 4th	4 (3–4)	Low	Regular	3 (3–3)
Medical rectal examination	Variable	5th	1 (1–1)	High	Regular	2 (1.5–2)
Mare reproductive rectal examination	Regular	5th	1 (1–1)	High	Regular	2 (2–2)

**Table 4:** The number of horse sessions and horse hours for each type of practical teaching class involving the use of horses during the 2018 calendar year and teaching semesters one and two.

	Calendar Year		Semester 1		Semester 2	
	Horse Sessions	Horse Hours	Horse Sessions	Horse Hours	Horse Sessions	Horse Hours
Animal handling	82 (15.4%)	192	31 (17.9%)	63	51 (19.6%)	129
Clinical examination	127 (23.8%)	358.5	45 (26%)	100	74 (28.5%)	240.5
Dental training	33 (6.2%)	102	10 (5.8%)	22	19 (7.3%)	70
Foot trimming	9 (1.7%)	25	9 (5.2%)	25	0 (0%)	0
Lameness evaluation	50 (9.4%)	155.5	23 (13.3%)	68.5	27 (10.4%)	87
Medical rectal examination	95 (17.8%)	176	19 (11%)	50	38 (14.6%)	62
Mare reproductive rectal examination	138 (25.8%)	267.5	36 (20.8%)	67.5	51 (19.6%)	94.5
TOTAL	534 (100%)	1276.5	173 (100%)	396	260 (100%)	683
